# Clinical Characteristics and Adverse Events in Acute Coronary Syndrome Patients with a History of Peripheral Arterial Disease

**DOI:** 10.5935/abc.20190150

**Published:** 2019-09

**Authors:** Yun-Peng Kang, Li-Ying Chen, Tie-Duo Kang, Wen-Xian Liu

**Affiliations:** Beijing An Zhen Hospital affiliated to Capital Medical University, Beijing - China

**Keywords:** Acute Coronary Syndrome, Atherosclerosis, Mortality, Peripheral Arterial Disease, Hospitalization/complications, Diabetes Mellitus, Risk Factors

## Abstract

**Background:**

In clinical observation, patients with acute coronary syndrome complicated with peripheral artery disease have poor prognosis, so the relationship between the diseases and clinical characteristics need to be further explored.

**Objective:**

This study aims to investigate clinical characteristics and independent risk factors for in-hospital adverse events in acute coronary syndrome patients with a history of peripheral arterial disease (PAD).

**Methods:**

A total of 5,682 patients with acute coronary syndrome were included into this study. These patients were divided into two groups according to the presence or absence of a history of PAD: PAD group (n = 188), and non-PAD (control) group (n = 5,494). Then, the clinical characteristics and incidence of in-hospital adverse events were analyzed; p < 0.05 was considered statistically significant.

**Results:**

The age of PAD patients was higher than that in the control group (65.5 ± 10.3 years vs. 58.6 ± 11 years, p < 0.001), and the proportion of PAD patients with diabetes history and stroke history was higher than that in the control group (73 [39%] vs. 1472 [26.8%], p = 0.018; 36 [19.3%] vs. 396 [7.2%], p < 0.001). The multivariate logistic regression analysis between groups based on in-hospital adverse events revealed that a history of PAD (OR = 1.791, p = 0.01), a history of diabetes (OR = 1.223, p = 0.001), and age of > 65 years old (OR = 4.670, p < 0.001) were independent risk factors for in-hospital adverse events.

**Conclusion:**

A history of PAD, advanced age, and a history of diabetes are independent risk factors for in-hospital adverse events in patients with acute coronary syndrome.

## Introduction

Atherosclerosis is a systemic vascular disease and one of the main causes of death and disability among Chinese residents. It mainly occurs in the coronary and cerebral arteries and affects the peripheral arteries (upper extremity, lower extremity, mesenteric and carotid arteries).

Peripheral artery disease (PAD) is a general name that refers to vascular diseases, except for cardio-cerebrovascular diseases. The narrow concept of PAD mainly refers to atherosclerotic stenosis or occlusion of the lower extremities, which causes symptoms of chronic or acute ischemia in the lower extremities.^1^ PAD patients have a high risk of cardiovascular disease. A study^2^ revealed that the risk of myocardial infarction in patients with PAD increased by 20-60%, and the risk of death caused by coronary artery disease (CAD) increased by 2-6 times. Therefore, similar to CAD), PAD can be a powerful predictor of death induced by myocardial infarction, stroke and other vascular diseases,^3^ and is closely correlated with the occurrence of death for cardiovascular events.^4^ Since the proportion of patients with lower extremity arterial disease is high in patients with PAD, in the present study, the lower extremity arterial disease was included into the concept of PAD and was investigated and discussed.

## Methods

Subjects: A total of 5,682 patients with acute coronary syndrome (ACS), who were admitted to the Department of Cardiology, Beijing Anzhen Hospital from April 2002 to August 2016, were included into the present study. Among these patients, 188 patients had a history of PAD. The age of the patients ranged from 36 to 84 years, with a median age of 64 years old; 143 patients were male (76.1%) and 45 patients were female (23.9%). The remaining 5,494 ACS patients without PAD were assigned as the control group. The age of these patients ranged from 25 to 90 years old, with a median age of 59 years old; 3,972 patients were male (72.3%) and 1,522 patients were female (27.7%).

Inclusion and exclusion criteria: Patients diagnosed and treated for ACS, with history of PAD were included into the study. The diagnostic criteria for ACS were based on the 2015 European Society of Cardiology diagnostic criteria.^5^

Exclusion criteria: patients with previous admissions for myocardial infarction, patients with acute myocardial infarction caused by embolus shedding, intravascular operation, or other diseases; patients who presented with cardiogenic shock, cardiac arrest and gastrointestinal bleeding upon admission; patients with acute infectious disease, malignant tumors and autoimmune diseases; pregnant women.

Diagnostic criteria for related diseases: diabetes mellitus was diagnosed based on the Guidelines for the Prevention and Treatment of Diabetes in China (2013 Edition).^6^ The criteria for diabetes diagnosis were based on the typical symptoms of diabetes in addition to random blood glucose ≥ 200 mg/dL and/or fasting blood glucose ≥ 126 mg/dL and/or blood glucose level at two hours after glucose load ≥ 200 mg/dL. Hypertension was diagnosed according to the Chinese Guidelines for the Management of Hypertension in China (2015 revised edition).^7^ The patient was diagnosed with hypertension when systolic blood pressure (SBP) was ≥ 140 mmHg (1 mmHg = 0.133 kPa) or diastolic blood pressure (SBP) was ≥ 90 mmHg. Dyslipidemia was diagnosed according to the Guidelines for Prevention and Treatment of Dyslipidemia in Chinese Adults (2016 revised edition):^8^ triglyceride (TG) ≥ 150 mg/dL, total cholesterol (TC) ≥ 201 mg/dL, low-density lipoprotein cholesterol (LDL-C) ≥ 131 mg/dL, high-density lipoprotein cholesterol (HDL-C) < 38 mg/dL, and smoking ≥ 10 cigarettes per day for more than one year.

Clinical data acquisition: (1) baseline clinical and demographical data of patients were recorded, including gender, age, body mass index, smoking history, alcohol consumption, family history of CAD, and past history of diabetes, hypertension, and dyslipidemia; (2) clinical indicators were recorded within 24 hours after admission, including heart rate, SBP and DBP. Fasting blood samples were collected in the morning of the next day after admission for the laboratory tests - blood routine test (complete blood count and platelet count) was performed using an automatic blood cell analyzer; blood lipid profile (triglyceride triacylglycerol, TC, LDL-C, and HDL-C) was determined using an automatic biochemical analyzer; brain (B-type) natriuretic peptide concentration was determined by radioimmunoassay, and troponin I was determined by mass spectrometry. The echocardiographic indexes included left ventricular ejection fraction (LVEF) and left ventricular end-diastolic diameter. The coronary angiography results were recorded after admission. In-hospital adverse events included acute left heart failure, cardiogenic shock, cardiac arrest and death.

### Statistical analysis

Statistical analysis was conducted using the statistical software SPSS 22.0. Normally distributed data were expressed as mean ± standard deviation, and non-normally distributed measurement data were expressed as median and interquartile range (P25, P75), and counts expressed as percentage. Data with normal distribution were compared using independent sample *t*-test, non-normally distributed continuous variables were evaluated using Mann-Whitney U-test, and discrete variables were compared using Chi-square (*X*^2^) test. The multivariate logistic regression analysis between groups for in-hospital adverse events was performed. A two-sided test was used in the present study, and a p < 0.05 was considered statistically significant.

## Results

Comparison of baseline data: mean age of PAD patients was 65.5 ± 10.3 years old and mean age of patients in control group was 58.6 ± 11 years old, with a statistically significant difference (p<0.05). The proportion of patients with diabetes mellitus in the PAD group was 39%, while that in the non-PAD group was 26.8%, and the difference was statistically significant. The analysis of clinical data after admission revealed that the levels of creatinine, TC and LDL-C were significantly higher in the PAD group than in the non-PAD (p < 0.05, for all; Table 1).

**Table 1:** Baseline data of acute coronary syndrome patients with and without a history of peripheral arterial disease admitted to hospital

Items	ACS with a history of PAD (n = 188)	ACS without a history of PAD (n = 5,494)	p
Age (years)	65.5 ± 10.3	58.6 ± 11.0	< 0.001
Male (%)	143(76.1)	3972(72.3)	0.472
History of hypertension (%)	123(65.9)	3175(57.8)	0.129
History of diabetes mellitus (%)	73(39)	1472(26.8)	0.018
Dyslipidemia (%)	24(12.5)	890(16.2)	0.464
History of smoking (%)	104(55.7)	3044(55.4)	0.987
History of alcohol intake (%)	30(15.9)	1170(21.3)	0.464
History of stroke (%)	36(19.3)	396(7.2)	< 0.001
SBP (mmHg)	126.36 ± 20.25	124.47 ± 26.67	0.389
DBP (mmHg)	72.47 ± 12.01	74.02 ± 13.03	0.233
HR (bpm)	76.09 ± 14.03	74.44 ± 19.37	0.280
WBC (10^9^/L)	7.3(5.9,9.7)	7.3(5.9,9.6)	0.801
RBC (10^12^/L)	4.3(3.9,4.6)	4.5(4.1,4.8)	0.001
PLT (10^9^/L)	205.04 ± 69.76	206.88 ± 66.03	0.795
ALT(U/L)	26.0(17.0,41.0)	26.0(17.0,44.0)	0.510
Creatinine (mg/dL)	0.97(0.75,1.24)	0.87(0.75,1.02)	0.021
AU (mg/dL)	5.91 ± 1.89	5.78 ± 2.13	0.545
Fasting plasma glucose (mg/dL)	106.3(93.7,147.7)	108.1(93.7,136.9)	0.381
TG (mmol/L)	123.9(79.7,159.4)	132.8(88.5,185.9)	0.079
TC (mg/dL)	166.3(139.2,189.5)	154.6(127.6,170.1)	0.002
LDL-C (mg/dL)	100.62(81.3,123.8)	89.0(73.5,108.4)	0.004
HDL-C (mg/dL)	34.8(27.1,46.4)	34.8(30.9,46.4)	0.586
D-dimer (umol/L)	99.0(50.0,196.2)	105.0(50.0,188.0)	0.832

Data expressed as mean ± standard deviation or median (interquartile range). ACS: acute coronary syndrome; PAD: peripheral arterial disease; SBC: systolic blood pressure; DBP: diastolic blood pressure; HR: heart rate; WBC: white blood count; RBC: red blood count; PLT: platelet count; ALT: alanine aminotransferase; AU: albumin in urine; TG: triglycerides; TC: total cholesterol; LDL-C: low density lipoprotein; HDL-C: high density lipoprotein.

Characteristics of the coronary artery: coronary angiography was performed for all included patients. Multi-vessel disease was defined as the presence of two or more main branches of the coronary artery or its major branches with ≥70% stenosis.^8,9^ According to these features, the disease was divided into three types: left main coronary artery disease (≥50% stenosis in the left main trunk), total occlusion (100% vascular stenosis), and calcification. The proportion of patients with multi-vessel stenosis was 12.1% in the PAD group, significantly higher than the non-PAD group (p < 0.05). In terms of left main CAD, occlusion, calcification and bifurcation lesions, the proportion of patients with these diseases was higher in patients with ACS combined with stroke, than in patients without stroke; but the difference was not statistically significant (Table 2).

**Table 2:** Characteristics of coronary artery lesions in acute coronary syndrome patients with and without a history of peripheral arterial disease [case(%)]

Items	ACS with a history of PAD (n = 188)	ACS without a history of PAD (n = 5494)	p
Left main coronary artery disease	9(4.8)	206(3.7)	0.777
Multivessel stenosis	22(12.1)	478(8.7)	0.015
Bifurcation lesion	27(14.4)	917(16.7)	0.782
Occlusion lesion	18(4.3)	191(3.6)	0.511
History of diabetes Calcified lesions	3(0.7)	15(0.2)	0.656

ACS: acute coronary syndrome; PAD: peripheral arterial disease.

Comparison of in-hospital adverse events: adverse events during in-hospital treatment included death, cardiogenic shock, acute left heart failure and cardiac rupture. In-hospital mortality rate was statistically significantly higher in ACS patients in the PAD group (1.1%), compared with patients in the control group (0.4%) (p < 0.05, Table 3).

**Table 3:** The incidence of adverse events in acute coronary syndrome patients with and without a history of peripheral arterial disease [case(%)]

Items	ACS merged with PAD history (n = 188)	ACS not merged with PAD history (n = 5,494)	p
Death	5(2.6)	23(0.4)	0.035
Cardiogenic shock	6(3.1)	203(3.7)	0.435
Acute left heart failure	7(3.7)	174(3.2)	0.355
Cardiac rupture	0(0)	2(0.03)	0.707

ACS: acute coronary syndrome. PAD: peripheral arterial disease.

After the patients were grouped according to the presence of the above events, the variables were selected for multivariate logistic regression analysis. The results revealed that history of PAD (OR = 1.791, p = 0.01), history of diabetes (OR = 1.223, p = 0.001), and age of >65 years old (OR = 4.670, p < 0.001) were independent risk factors for in-hospital adverse events (Table 4).

**Table 4:** Multivariate Logistic regression analysis based on in-hospital adverse events

Items		SE		OR		95%CI		p
History of PAD		0.220		1.791		1.05-2.88		0.010
History of hypertension		0.169		1.112		0.79-1.55		0.529
History of diabetes mellitus		0.082		1.223		1.01-1.41		< 0.001
Age > 65 years old		0.181		4.670		3.21-6.44		< 0.001
Multivessel disease		1.015		0.625		0.08-4.57		0.643

PAD: peripheral arterial disease.

## Discussion

There is a close correlation between atherosclerotic heart disease and PAD.^10^ Patients with PAD have more extensive atherosclerosis, and the lesions are often more serious. Therefore, the risk of atherosclerotic events in this group is further increased. In the GRACE trial, approximately 9.7% of 41,108 ACS patients had PAD.^11^ In the present study, 5,682 ACS patients were included; 188 of them (3.3%) had a history of PAD, and this proportion was lower than the proportion reported in the GRACE trial.

The occurrence and development of atherosclerosis are closely correlated to age. A study revealed that PAD patients were older and had higher risk of cardiovascular disease.^12^ Furthermore, the present study revealed that patients with ACS complicated with PAD was older than patients in the control group. The multivariate logistic regression analysis of in-hospital adverse events between the groups revealed that an age of ≥65 years old is an independent risk factor for in-hospital adverse events (OR = 4.670, p < 0.001). The older the age, the higher the incidence of the adverse events, which is consistent with existing literature. Therefore, for elderly ACS patients with a history of PAD, more attention should be given to changes in patient condition. In the present study, although the incidence of stroke in ACS patients with a history of PAD was higher (19.3%) than in controls, further analysis revealed that it did not affect in-hospital adverse events.

Dyslipidemia has been considered an important risk factor for atherosclerosis.^13^ Existing studies have shown that cholesterol is closely related to the occurrence and development of PAD. In the formation and development of atherosclerosis, LDL-C plays an important role. Furthermore, evidence supporting the relationship between LDL-C and PAD has been found.^14^ The present study also revealed that the level of LDL-C was higher in the PAD group than in the control group. Therefore, lipid-lowering therapy should be strengthened for patients with PAD complicated with ACS. Among the common risk factors for atherosclerosis, diabetes has been well-recognized as an independent risk factor for atherosclerosis. The ARIC study^15^ revealed that, as compared with patients with a 0-5-year course of diabetes, the risk for PAD in patients with a course of diabetes of ≥ 6 years significantly increased, and the relative risk was 1.24. The present study also revealed that a history of diabetes was an independent risk factor for in-hospital adverse events (OR = 1.223, p < 0.001). Long-term hyperglycemia affects the elasticity and stiffness of the blood vessel walls, which leads to endothelial dysfunction and microcirculatory dysfunction. Therefore, controlling blood sugar is a necessary measure to reduce the incidence of ACS and PAD.^16^

Analysis of the characteristics of coronary arterial lesions revealed that compared with left main coronary artery disease, bifurcation lesions and calcification, and other serious CADs, ACS patients with PAD are more frequently affected by multivessel disease in coronary arteries. Also, atherosclerosis was characterized by extensive vascular involvement in coronary arteries. Therefore, multivessel lesions tend to indicate extensive wall motion abnormalities, leading to poor prognosis. This study has also confirmed this.

In the present study, analysis of in-hospital adverse events revealed that in-hospital mortality in ACS patients with PAD was 1.1% (p = 0.035), and the difference was statistically significant when compared with the control group. A meta-analysis revealed that after 2.7 years of follow-up for patients with acute myocardial infarction complicated with a PAD history, cardiovascular deaths occurred in 17.8% of patients, and 52.3% of these patients, and only 28% of patients without PAD experienced re-hospitalization caused by nonfatal myocardial infarction, nonfatal stroke and heart failure. Therefore, PAD is an independent risk factor for predicting poor outcomes.^17^

Thus, it can be concluded that patients with a history of PAD are more likely to experience many adverse events. In the present study, mortality due to adverse events was lower than that reported in the literature. The reasons for these differences may be that the subjects included in the present study were ACS patients, including low-risk patients such as patients with unstable angina. Furthermore, patients were not followed-up, and only in-hospital cardiovascular deaths were counted.

The limitation of the present study was that it had a single-center, retrospective design, and a single-center study may have bias in case selection. Furthermore, our sample (ACS population) included patients with unstable angina, non-ST-segment elevation myocardial infarction and ST-segment elevation myocardial infarction. The difference in severity between these conditions may have led to observation bias.

## Conclusion

Patients with ACS complicated with a history of PAD have extensive coronary disease and high in-hospital mortality. A history of PAD is an independent risk factor for in-hospital adverse events.
